# Predictive value of APAF-1 and COX-2 expression in pathologic complete response to neoadjuvant chemoradiotherapy for patients with locally advanced rectal adenocarcinoma

**DOI:** 10.18632/oncotarget.9125

**Published:** 2016-05-02

**Authors:** Haihua Peng, Kaiyun You, Rong Zhang, Shaoyan Xi, Tian Zhang, Jun Dong, Muyan Cai, Chengtao Wang, Huizhong Zhang, Tongchong Zhou, Yuanhong Gao, Bixiu Wen

**Affiliations:** ^1^ Department of Radiation Oncology, The First Affiliated Hospital of Sun Yat-sen University, Guangzhou 510080, China; ^2^ Department of Radiation Oncology, SunYat-Sen University Cancer Center, State Key Laboratory of Oncology in Southern China, Guangzhou 510060, China; ^3^ Department of Endoscopy and Laser, Sun Yat-sen University Cancer Center, State Key Laboratory of Oncology in South China, Collaborative Innovation Center for Cancer Medicine, Guangzhou 510060, China; ^4^ Department of Pathology, Sun Yat-sen University Cancer Center, State Key Laboratory of Oncology in South China, Collaborative Innovation Center for Cancer Medicine, Guangzhou 510060, China; ^5^ Department of Radiation Oncology, Cancer Center of Guangzhou Medical University, Guangzhou 510075, China; ^6^ Department of Radiation Oncology, Sun Yat-sen Memorial Hospital, Sun Yat-sen University, Guangzhou 510120, China

**Keywords:** rectal adenocarcinoma, neoadjuvant chemoradiotherapy, complete pathological response, APAF-1, COX-2

## Abstract

**Purpose:**

To investigate predictive value of APAF-1 and COX-2 expression in pathologic complete response (pCR) for patients with rectal adenocarcinoma (RAC) who were treated with neoadjuvant chemoradiotherapy (neo-CRT) followed by total mesorectal excision (TME).

**Materials and Methods:**

Immunohistochemistry assay was used to detect expression of APAF-1 and COX-2 in paraffin-wax embedded tissues obtained before neo-CRT for patients with RAC. A 5-point tumor-regression grade (TRG) based on the ratio of residual tumor to fibrosis according to Dworak's scoring system was used to assess neo-CRT response. The relationship between expression of APAF-1 and COX-2 genes and pCR was explored.

**Results:**

pCR (TRG4) was observed in 23 patients (28.0%). pCR were more likely to be achieved for those with APAF-1 over-expression or lower expression of COX-2. pCR rate in patients with combination of high APAF-1 and low COX-2 expression was 56.0%, significantly higher than those with other combination of APAF1 and COX-2 expression. Multivariate analysis showed that over-expression of APAF-1 and suppressed expression of COX-2 were independent predictive factors for pCR.

**Conclusion:**

Immunohistochemical evaluation of APAF-1 and COX-2 expression on pretreatment specimen may be used to predict pCR to neo-CRT in patients with RAC. The potential of the markers in monitoring pCR patient merits further investigation.

## INTRODUCTION

Neoadjuvant chemoradiotherapy (neo-CRT) followed by total mesorectal excision (TME) is the standard of treatment for patients with locally advanced rectal adenocarcinoma. neo-CRT results in varying degrees of tumor regression that range from pathologic complete response (pCR) to modest or no treatment response. Studies have demonstrated that patients with pCR to neo-CRT have shown better prognosis than those with non-pCR [1]. There is a trend that patients who present pCR to neo-CRT might receive local excision [2, 3] or wait-and-see policy [4] to avoid radical surgery related complications. The key to clinical follow-up study is to establish an accurate and accepted model to screen those who may achieve pCR after neo-CRT and before surgery.

Currently, researchers have extensively explored the clinicopathologic factors and molecular markers in predicting pCR to neo-CRT in locally advanced rectal adenocarcinoma. It has been discovered that pre-treatment serum albumin > 3.5 mg/ml, the ratio of neutrophils/lymphocytes < 5 and percent of circulating blood lymphocytes were closely associated with pCR [5] and that pre-treatment hemoglobin (Hb) level could be used to predict not only pCR to neo-CRT but also local tumor recurrence [6]. Other factors such as tumor size, CEA and clinical N stage were also investigated to predict pCR to neo-CRT. Whereas studies have shown that molecular markers such as p53, Ki-67, Bcl-2/Bax cannot be used to predict the tumor response to chemoradiotherapy. Although EGFR, thymidylate synthase, p21 have been reported to be associated with chemoradiotherapy response, further evidence is still warranted [7].

Tumor growth and metastasis depend on angiogenesis; while COX-2 has been reported to be closely associated with cell proliferation and angiogenesis. Apoptosis protease-activating factor 1 (APAF-1) is a key regulator in mitochondrial apoptotic pathway and radiation-induced apoptosis is believed to be the main form of cancer cell death caused by radiotherapy. In this study, we are going to detect expression of COX-2 and APAF-1 genes in tissue samples obtained from pretreatment specimen for patients with locally advanced rectal adenocarcinoma and explore their potential value in predicting treatment response especially pCR to neo-CRT.

## RESULTS

### Pathologic tumor response to neo-CRT and its association with clinicopathologic features

The tumor response to neo-CRT according to pathological evaluation of tissue samples after TME was reported as TRG 0 in none, TRG 1 in 6 (7.3%), TRG 2 in 33 (40.2%), TRG 3 in 20 (24.4%) and TRG 4 (pCR) in 23 (28.0%), respectively. The relationship between the status of pCR and clinicopathologic factors see Table S1.

### Immunohistochemical staining for expression of APAF-1 gene

**Figure 1:**
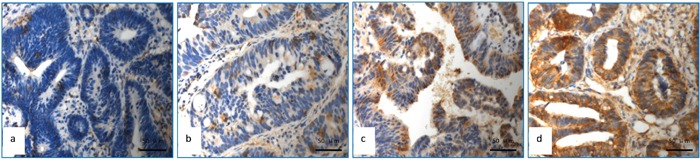
Detection of APAF-1 expression using immunohistochemical assay **a.** APAF-1with no expression; **b.** APAF-1 low expression (Express degree, 1 score = light yellow granules, 1 score × number of positive tumour cells 25%, 1 score); **c.** APAF-1 high expression (Express degree, 6 score = brown granular, 3 score×number of positive tumour cells 50%, 2 score); **d.** APAF-1 high expression (Express degree, 9 score = brown granular, 3 score×number of positive tumour cells 90%, 3 score).

The representative data for immunohistochemical staining of APAF-1 gene expression were shown in Figure 1. The expression score for APAF-1 was reported as 1 point in 4 patients, 2 points in 9 patients, 3 points in 11 patients, 4 points in 12 patients, 6 points in 27 patients and 9 points in 19 cases, respectively (Seen in Table 1). Fisher test was used to explore each expression rate with pathological correlation of pCR. 4 points was used as demarcation point of APAF-1 gene expression. Expression score > 4 points was defined as high expression and ≤4 points as low expression (Figure 1a-1d). In the group of APAF-1 high expression, 17 cases were shown to achieve pCR (37.0%) which was significantly higher than those in group of APAF-1 low expression (16.7%)(p=0.042) (Table 2). Detailed analysis did not show significant correlation between the expression level of APAF-1 and clinicopathologic factors except high level of APAF-1 expression in cN0 patients(Table S2).

**Table 1:** The degree of APAF-1 expression in the whole group

Expression score	n	pCR (n)	Non-pCR (n)
1	4	1	3
2	12	2	10
3	11	1	10
4	9	2	7
6	27	12	15
9	19	5	14
Total	82	23	59

**Table 2:** Relationship between cut-off of APAF-1 and COX-2 and pCR

Expression score	pCR (n)	non-pCR (n)	*p* value
APAF-1 Cut-off of 4			0.042
1-4 (low)	6	30	
5-9 (high)	17	29	
COX-2 Cut-off of 6			0.024
1-6 (low) vs	18	30	
7-9 (high)	5	29	

### Immunohistochemical staining for expression of COX-2 gene

**Figure 2:**
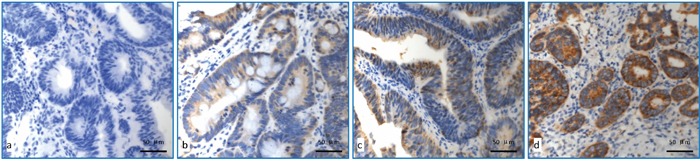
Detection of COX-2 expression using immunohistochemical staining **a.** COX-2 with no expression; **b.** COX-2 low expression (Express degree, 1 score = light yellow granules, 1 score×number of positive tumour cells 25%, 1 score); **c.** COX-2 moderate expression (Express degree, 6 score = brown granular, 3 score × number of positive tumour cells 50%, 2 score); **d.** COX-2 high expression (Express degree, 9 score = brown granular, 3 score × number of positive tumour cells 95%, 3 score).

The representative data for immunohistochemical staining of COX2 gene expression were shown in Figure 2. The detailed score for COX-2 expression was shown in Table 3. To explore each expression rate with pathological correlation of pCR using fisher test, 6 points was used as demarcation point, expression score > 6 points is defined as high expression and ≤6 points as low expression. For patients with low expression of COX-2, 18 cases (37.5%) achieved pCR, which was significantly higher than those with COX-2 high expression (14.7%)(p=0.024)(Table 2). No significant correlation between the expression level of COX-2 and clinicopathologic factors was observed. (Table S3).

**Table 3:** The degree of COX-2 expression in the whole group

Expression score	N	pCR (n)	No-pCR (n)
1	1	0	1
3	1	0	1
4	1	0	1
6	45	18	27
9	34	5	29
Total	82	23	59

### Tumor response to neo-CRT and its association with combined expression of APAF-1 and COX-2 genes

**Figure 3:**
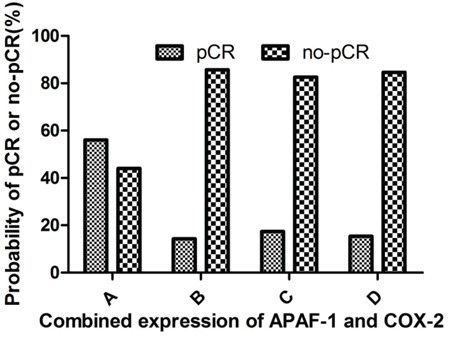
Correlation between the pCR and combined expression of APAF-1 and COX-2 genes **A.** high APAF-1/low COX-2 expression group; **B.** high APAF-1/high COX-2 expression group; **C.** low APAF-1/low COX-2 expression group; **D.** low APAF-1/high COX-2 expression group. The probability to achieve pCR rate was 56.0% for patients with high APAF-1/low COX-2 expression, significantly higher than those with high APAF-1/high COX-2 expression group (14.3%), low APAF-1/low COX-2 expression group (17.4%) and low APAF-1/high COX-2 expression group 15.4% (p=0.005).

Analysis of combined APAF-1 and COX-2 gene expression in predicting pCR showed that patients with high expression of APAF-1/low expression of COX-2 were associated with achieving the highest pCR rate (56.0%), which was significantly higher than those with high expression of APAF-1/high expression of COX-2 (14.3%), low expression of APAF-1/low expression of COX-2 (17.4%), low expression of APAF-1/high expression of COX-2 (15.4%) (p=0.005) (Table 4, Figure 3).

**Table 4:** Correlation between pCR and combined expression ofAPAF-1and COX-2

Group	APAF-1	COX-2	No.	pCR (%)	no-pCR (%)
A	High	Low	25	14 (56.0%)	11 (44.0%)
B	High	High	21	3 (14.3%)	18 (85.7%)
C	Low	Low	23	4 (17.4%)	19 (82.6%)
D	Low	High	13	2 (15.4%)	11 (84.6%)

### Univariate logistic analyses of predictors for pCR to neo-CRT

As shown in Table 5, univariate logistic analysis was demonstrated that both APAF-1 and COX-2 gene expression were independent risk factors for pCR after neo-CRT. Other clinical factors including age, gender, histological grade, tumor distance from anal verge, clinical stage and adjuvant chemotherapy were not associated with tumor response to neo-CRT except the serum carcino-embryonic antigen (CEA). The median CEA levels prior to neo-CRT was 2.43 ng/ml (0.69-10.90 ng/ml) in the group of pCR (TRG 4) and 4.38 ng/ml (0.57-206.20 ng/ml) in the group of non-pCR (TRG 0-3), the difference was statistically significant (p=0.001).

**Table 5:** Uni- and multi-variate logistic analysis of clinicopathologic factors for pCR

Variables	Univariate		Multivariate	
	HR (95% CI)	p value	HR (95% CI)	p value
Age (year)				
≤56 vs >56	1.129 (0.430-2.960)	0.806	—	—
Gender				
Male vs female	0.743 (0.253-2.186)	0.590	—	—
Hg (g/L)				
≤110 vs >110	4.490 (0.541-37.27)	0.164	—	—
Tumor location (cm)				
≤5.0 vs >5.0	0.762 (0.285-2.033)	0.587	—	—
CEA level ( ng/ml)				
<5.00 vs ≥5.00	0.378 (0.124-1.155)	0.088	0.300 (0.089-1.012)	0.052
Histologic grade				
G1-2 vs G3	0.983 (0.306-3.156)	0.977	—	—
APAF-1expression				
low vs high	2.931 (1.014-8.473)	0.047	4.291 (1.342-13.699)	0.014
COX-2 expression				
low vs high	0.287 (0.094-0.876)	0.028	0.205 (0.059-0.708)	0.012
Interval between completion of RT and surgery (weeks)#					
≤7 vs >7	0.695 (0.263-1.833)	0.462	—	—

HR: hazard ratio; CI: confidence interval;

# interval between neo-CRT and surgery

### Multivariate logistic analyses of predictors for pCR to neo-CRT

Multivariate logistic analyses were shown that both APAF-1 and COX-2 gene expression remained to be independent risk factors for pCR. Pre-CRT serum CEA level was not associated with predicting pathologic tumor response to neo-CRT (p=0.052) (Table 5).

## DISCUSSION

Our research has shown that evaluation of APAF-1 and COX-2 expression on pretreatment specimen may be used to predict pathologic complete response to neo-CRT in patients with locally advanced rectal adenocarcinoma. We and other researchers have demonstrated that pathologic stage is strongly associated with treatment outcome for thosewho were treated with neoadjuvant chemoradiotherapy followed by radical surgery, especially for those with pCR after neo-CRT can achieve a better prognosis [8, 9]. The ability to monitor pCR patients after neo-CRT before radical surgery would significantly impact subsequent management. Patients who would achieve pCR after neo-CRT may be recommended to have local excision or wait and see treatment strategy to avoid radical surgery-related sequelae and complications [10].

Researchers have mainly focused on the clinical factors, imaging studies and molecular targets to monitor pCR patients after neo-CRT. Our research have shown that clinical factors such as age, gender, hemoglobin (Hb) and histological grade were not associate with pCR [11]. Yet Khan A et al [6] have demonstrated that Hb might be used as a biomarker to predict treatment response of rectal adenocarcinoma to neo-CRT. Garcia-Florez et al have reported that the differentiation level of tumor cells can be used to predict treatment response to neo-CRT in patients with rectal adenocarcinoma [12]. Perez et al [13] have shown that assessment of treatment response with PET/CT imaging at 12 weeks after the completion of CRT may provide additional information for the accurate selection of patients achieving complete clinical response who may avoid unnecessary radical resection. Peng HH et al [14] have reported that transrectal ultrasonography (TRUS) restaging has potential value in screening patients with pCR in 149 patients with locally advanced rectal adenocarcinoma after neo-CRT when compared to postoperative pathological TNM staging.

APAF-1 is a key regulating factor of mitochondrial apoptosis pathway. It can combine with cytochrome C, Caspase–9 to form an apoptotic body and therefore activate caspase execution system leading to programmed cell death [15]. Zlobec et al [16] have reported that detection rate of APAF-1 expression for patients who achieved pCR, partial response and no response after neo-CRT was 60%, 51.0% and 27.6%, respectively. Therefore they have concluded that high level of pretreatment APAF-1 expression indicated higher rates of pCR that would be expected for paitents with locally advanced rectal adenocarcinoma when they were treated with neo-CRT. Edden Y et al [17] have also discovered that the level of APAF-1 expression was significantly associated with tumor regression, T downstage and pCR and concluded that APAF-1 was one of the independent factors to predict tumor regression to neo-CRT. Yet Garcia-Florez [12] have made an opposite conclusion that high expression of APAF-1 lead to lower pCR in locally advanced rectal adenocarcinoma after neo-CRT.

COX-2 is the critical enzymes involved in transformation of arachidonic acid into prostaglandins. It works through promoting the production of prostaglandins, which may in turn enhance cell proliferation, promote the formation of blood vessels, inhibit cell apoptosis and exert anti-tumor immune response [18, 19]. Kishi et al [20] have confirmed in nude mouse sarcoma model that COX-2 inhibitors can reduce the expression of prostaglandin E2, suppress tumor angiogenesis, thus inhibit tumor growth. Clinical studies in patients with locally advanced rectal adenocarcinoma have shown that high COX-2 expression conveys poorer sensitivity to radiation and chemotherapy by promoting high expression of angiogenesis factor [21], and using the COX-2 inhibitor celecoxib can improve the sensitivity of tumor cells to radiation therapy [22].

In this study we have focused on the relationship between pCR after neo-CRT and expression level of APAF-1and COX-2 genes in locally advanced rectal adenocarcinoma. Our research data have demonstrated the positive predictive value of a strong APAF-1 pretreatment expression for patients to achieve pCR after neo-CRT and an inverse correlation between increased level of COX-2 and pCR, which were quite similar to the reports [17].

To the best of our knowledge, it is the first research to explore the predicting value of combined expression of APAF-1 and COX-2 genes in pCR to neo-CRT in patients with locally advanced rectal adenocarcinoma. Our data have demonstrated that patients with high expression of APAF-1/low expression of COX-2 would be expected to obtain the highest pCR rate (56.0%), which is significantly higher than those with other combination of their expression, which might be used to monitor pCR patients before neo-CRT.

There may be several factors confounding the results in this study. Firstly it was a retrospective study. We only selected those with preoperative biopsy paraffin blocks available and matched TME surgical specimens. Secondly the sample size was relatively small with only 82 patients included in this study.

In conclusion, the status of APAF-1 and COX-2 expression detected in pretreatment rectal tumor biopsies may be predictive in treatment response to neo-CRT for patients with locally advanced rectal adenocarcinoma. The combination of high expression of APAF1 and low expression of COX-2 might be used in selecting patients with pathologic complete response to neo-CRT. The potential of these two markers used to predict pCR merits further investigation.

## MATERIALS AND METHODS

82 consecutive patients with pre-treatment rectal biopsy paraffin tissue blocks and matched TME surgical specimens were treated with neo-CRT and TME from 2005 to 2012. 16 patients (19.5%) presented with stage II and 66 (80.5%) with stage III rectal adenocarcinoma in which pretreatment biopsy paraffin blocks were available for the evaluation of APAF1 and COX2 gene expression and TME surgical specimens for the evaluation of treatment response to neo-CRT. Among them 57 patients were (69.5%) male and 25 (30.5%) female, and the median age was 57 years (range: 15-75). All patients were made clinical and preoperative staging examination including digital rectal exam, chemistry profiling, colonoscopy, endorectal ultrasound, abdominal and pelvic CT and/or MRI, chest radiography.

Concurrent chemoradiotherapy was administered to all patients. Target volume was defined according to the recommendations of the ICRU reports No. 50 and 62 and Myerson RJ et al [23]. The gross tumour volume (GTV) was delineated further according to the information obtained from the diagnostic CT and MRI, including the rectal primary tumor and invaded lymph nodes. Two clinical target volumes (CTVs) were defined: CTV1 was the GTV plus the corresponding mesorectum and presacral region plus a margin of 2-5 cm in the cranio-caudal direction. CTV2 included the whole rectum and loco-regional lymph nodes at risk of involvement, the posterior part of prostate and seminal vesicles in male patients and the posterior of vaginal wall and cervix in female patients. The uppermost border for CTV2 was at the bifurcation of abdominal aorta approximated the sacral promontory and its lowermost border was at the anal verge covered the rectosigmoid junction and the whole rectum with its mesentery. PTV1, PTV2 were obtained by adding non-uniform margins to CTV1, CTV2 as below: the margins of the cranio-caudal, the anterior and posterior, and the lateral were 0.9cm, 0.7cm and 0.8cm, respectively. The organs at risk (OAR) volumes were outlined in the small bowel, the bladder, and the femoral heads. Radiotherapy with 50 Gy to the rectum as clinical tumor volume, CTV1) and 46 Gy was administered to the region of pelvic lymph node as clinical tumor volume, CTV2) in 1.8-2.0 Gy/fraction over a period of 5 weeks. Once the treatment planning was completed, the plan was normalized to cover 100% of the PTVs with ≥ 95% of the prescribed dose.

One of two chemotherapeutic regimens was delivered concurrent with RT: (1) FOLFOX: fluorouracil 3.0 g/m^2^, CIV lasting for 48 h; calcium folinate 200.0 mg/m^2^, day 1; oxaliplatin 100.0 mg/m^2^, day 1; repeated for three weeks (n=6 patients, 7.3%); and (2) XELOX: capecitabine 1000.0 mg/m^2^ bid, days 1–14; oxaliplatin 100.0 mg/m^2^, day 1; repeated for three weeks (n = 76 patients, 92.7%).

Surgery was performed approximately 7 weeks (range: 4-20 weeks) after the completion of neo-CRT. The surgical procedure was either low anterior resection/double stapling method (n=54 patients, 65.9%) or abdominoperineal resection (n = 28 patients, 34.1%).

52 patients received post-operative adjuvant chemotherapy with either XELOX (63%) or FOLFOX (34%). The regimen of adjuvant chemotherapy was consistent with the pre-operative chemotherapy with median cycle of 3 (range: 2–6) cycles.

### Immunohistochemical assay

Paraffin block from the pretreatment biopsy sample were sectioned at 3 μm in thickness, placed on positively charged slides. The immunohistochemical staining was made according to the recommendation by the company. The tissue sections were stained with the following antibodies: COX-2 (1:100, ABGENT, USA) and APAF-1 (1:100, ABGENT, USA). Negative control was made by substituting PBS for the primary antibodies.

### Analysis of immunohistochemical assay

The results of immunohistochemical staining for COX-2 and APAF-1 gene expression was evaluated by two independent pathologists blinded to the tumor response grade (TRG) to neo-CRT and final pathological staging. As recommended by Edden Y and Smith FM [17, 24], the intensity of staining and the number of positive tumor cells were used to classify cytoplasmic markers based on the following criteria: 0, none; 1, light yellow granules; 2, yellow granules; 3, brown granular. Number of positive tumor cells: 0, no staining; 1, up to 25% of positive cells; 2, 26–50% of positive cells; and 3, more than 50% of positive cells. The express degree score = Intensity of staining score × Number of positive tumor cells score.

The histological sections were originally examined by two experiencedpathologists independently. TRG was assessed by utilizing Dworak's scoring system [25] which defined the amount of residual carcinoma in relation to fibrosis on a five-point scale as follows: TRG 0, no regression; TRG 1, tumor regression less than 25%; TRG 2, tumor regression between 25%-50%; TRG 3, tumor regression more than 50% with fibrosis outgrowing the tumor mass; TRG4, complete pathologic response, only fibrosis (pCR).

### Statistical analysis

SPSS v17.0 software was used for statistical analysis. Classification variables were analyzed using chi-square test or fish precise inspection, quantitative variables using student t test or rank and inspection. Kaplan Meier method was adopted for survival analysis, and the log rank test was used between variables, and a *p* value < 0.05 was considered to be significant.

## SUPPLEMENTARY TABLES


