# Supplementary material for: Predictive value of APAF-1 and COX-2 expression in pathologic complete response to neoadjuvant chemoradiotherapy for patients with locally advanced rectal adenocarcinoma
Source: Oncotarget. 2016 May 2;7(23):35233–40. doi: 10.18632/oncotarget.9125 (PMC5085224; doi:10.18632/oncotarget.9125)
Supplement: Supplementary file 1 [file oncotarget-07-35233-s001.pdf]

Predictive value of APAF-1 and COX-2 expression in pathologic complete response to neoadjuvant chemoradiotherapy for patients with locally advanced rectal adenocarcinoma

SUPPLEMENTARY TABLES

Supplementary Table S1: The relationship between the status of pCR and clinicopathologic factors

Clinical factor	Number	pCR	no-pCR	*p* value
Age (years)				0.806
≤56	41	12	29	
>56	41	11	30	
Gender				
Male	57	17	40	0.585
Female	25	6	19	
Hb (g/L)				0.099
≤110	11	1	10	
>110	71	22	49	
CEA (ug/mL)				0.074
<5.00	52	18	34	
≥5.00	30	5	25	
Tumor location				0.586
>5.0cm	46	14	32	
≤5.0cm	36	9	27	
Clinical T stage				0.888
T1-2	4	1	3	
T3-4	78	22	56	
Clinical N stage				0.100
N0	16	2	14	
N+	66	21	45	
Histologic grade				0.977
G1-2	64	18	46	
G3	18	5	13	

Supplementary Table S2: The relationship between the expression level of APAF - 1 and the clinicopathologic factors

Clinical factor	Number	Low expression	High expression	p value
Age (years)				0.182
≤56	41	15	26	
>56	41	21	20	
Gender				
Male	57	22	35	0.144
Female	25	14	11	
Hb (g/L)				0.052
≤110	11	8	3	
>110	71	28	43	
CEA (ug/mL)				0.316
<5.00	52	25	27	
≥5.00	30	11	19	
Tumor location				0.075
>5.0cm	46	16	30	
≤5.0cm	36	20	16	
Clinical T stage				0.627
T1-2	4	1	3	
T3-4	78	35	43	
Clinical N stage				0.026
N0	16	11	5	
N+	66	25	41	
Histologic grade				0.958
G1-2	64	28	36	
G3	18	8	10	

Supplementary Table S3: The relationship between the expression level of COX-2 and the clinicopathologic factors

Clinical factor	Number	Low expression	High expression	p value
Age (years)				0.370
≤56	41	26	15	
>56	41	22	19	
Gender				0.200
Male	57	36	21	
Female	25	12	13	
Hb (g/L)				1.000
≤110	11	7	4	
>110	71	41	30	
CEA (ug/mL)				0.468
<5.00	52	32	20	
≥5.00	30	16	14	
Tumor location				0.658
>5.0cm	46	28	18	
≤5.0cm	36	20	16	
Clinical T stage				1.000
T1-2	4	2	2	
T3-4	78	46	42	
Clinical N stage				0.355
N0	16	11	5	
N+	66	37	29	
Histologic grade				0.771
G1-2	64	38	26	
G3	18	10	8	
